# Patient Experiences of a Theory-Based Lifestyle-Focused Group Treatment in the Prevention of Cardiovascular Diseases and Type 2 Diabetes

**DOI:** 10.1007/s12529-012-9252-3

**Published:** 2012-07-27

**Authors:** Sofia Ljung, Cecilia Olsson, Merith Rask, Bernt Lindahl

**Affiliations:** 1 Department of Food and Nutrition, Umeå University, Umeå, Sweden; 2 Behavioral Medicine, Umeå University Hospital, Umeå, Sweden; 3 Department of Public Health and Clinical Medicine, Umeå University, SE-901 87 Umeå, Sweden

**Keywords:** Behavioral medicine, Lifestyle, Self-efficacy, Prevention, Qualitative interviews, Patient experience

## Abstract

**Background:**

Cardiovascular disease and type 2 diabetes are two of the most common public health diseases, and up to 80 % of the cases may be prevented by lifestyle modification. The physiological effects of lifestyle-focused treatment are relatively well studied, but how patients actually experience such treatments is still rather unclear.

**Purpose:**

The aim of this study was to explore how patients experience lifestyle-focused group treatment in primary and secondary prevention of cardiovascular disease and type 2 diabetes.

**Method:**

Qualitative interviews were conducted with 19 patients attending lifestyle-focused group treatment based on social cognitive theory at a behavioral medicine clinic in northern Sweden. The interviews were transcribed verbatim and analyzed according to Malterud’s systematic text condensation.

**Results:**

The study shows that patients participating in this kind of group-based lifestyle treatment went through a process of self-development which deepened their understanding of own responsibility for health and improved their skills in finding support in others. The process could be tracked through three different themes (the holistic view, personal responsibility, and group treatment) which together reflected the most essential parts of the informants’ experience and showed the patient as an active decision maker struggling to adopt the principles of behavioral change.

**Conclusion:**

Lifestyle-focused group treatment, based on social cognitive theory, was shown to stimulate different components that strengthen patients’ self-efficacy for long-term behavioral change.

## Introduction

According to the World Health Organization, cardiovascular diseases have become the leading cause of death and were associated with 30 % of all deaths worldwide in 2008 [1]. Coincidentally, diabetes mellitus is expected to increase more than double, from 171 million in 2000 to 366 million in 2030 [2]. Main risk factors for these diseases are directly related to lifestyle, and up to 80 % of the morbidity could be prevented if lifestyle-related risk factors—such as physical inactivity, tobacco use, and poor eating habits—were eliminated [3]. Behavior modification has during the last decade been the standard for an intensive lifestyle program in primary prevention of type 2 diabetes [4, 5]. Increased physical activity and lowered energy intake, often based upon a low-fat high-fiber diet, have been shown to induce a multitude of beneficial effects, protecting people against type 2 diabetes [6]. Often a weight loss of 5–10 % of initial body weight is needed in order to generate positive effects on the metabolism [7]. In the Finnish Diabetes Prevention Study, a difference in weight loss between the intensive lifestyle group and the control group of 2.7 kg resulted in a reduction of diabetes development of 58 % [4].

The study of mechanisms behind behavioral change is facilitated when the intervention is theory-based. The theory, with all of its compounds and constructs, can be tested repeatedly in the same as well as in a different setting, making it easier to understand why one type of intervention will be successful while another is a failure [8]. Social cognitive theory (SCT) is one of the most used behavioral change theories, comprehensive in its nature and incorporating many different concepts [9]. According to SCT, there is a continuous ongoing interaction between the environment, the person, and the behavior. If the outcome of a behavioral change is appraised as something positive, then the new behavior will continue (outcome expectancy). If appraised as negative, the new behavior will disappear. However, even more important is a person’s self-efficacy for a specific behavior, i.e., the confidence a person has in his ability to perform the behavior (self-efficacy expectancy) [10].

An essential part of evaluating the effects of a health behavioral change program is to capture patient experiences, since it is their understanding of different program components that affects health behavioral change. Multiple studies have examined the physiological effects of lifestyle-focused treatments [11, 12], and some studies have also compared the effects of lifestyle-focused group treatments with similar individual treatments [13–15]. A few studies have investigated how patients experience individual lifestyle treatment [16–18], but to our knowledge, there are no specific studies that have examined patients’ experiences of lifestyle-focused group treatment in the prevention of cardiovascular disease and type 2 diabetes.

## Objective

The study aimed to explore how patients experience a theory-based lifestyle-focused group treatment in the prevention of cardiovascular disease and type 2 diabetes.

## Methods

### Study Population

The study population consisted of patients who participated in, or recently had completed, a theory-based lifestyle-focused group treatment at a behavioral medicine clinic in northern Sweden. The theoretical framework of the treatment at the clinic was based on SCT with emphasis on strengthening self-efficacy [9, 10]. The treatment focused on patients making small, measurable changes instead of having goals set to a very high level. The staff approached patients in a nondirective way, using the techniques of motivational interviewing and strived throughout the treatment to offer encouragement and support. The treatment included practical training in cooking, exercise, and stress management as well as lectures and group sessions about lifestyle-focused behavioral change. Prior to treatment, all patients had a BMI >30 and were at risk of developing, or had developed, type 2 diabetes and/or cardiovascular disease. Treatment began with two registration days which included weighing, measuring, taking blood samples, and meeting staff. After that, 1 month of self-preparation followed at home before a 10-day course of intensive treatment at the clinic. During the treatment year, four 2-day reunions were conducted, and after that, annual half-day follow-ups were carried out for another 3 years.

### Sample

Initially, an invitation letter was sent to 88 patients, in eight treatment groups, which all differed regarding treatment time. Patients who had dropped out of treatment were excluded from the invitation. Since only five patients responded, the recruitment method was modified and an invitation letter was instead handed out to 42 patients, in six treatment groups, who visited the clinic in February to March 2011. Overall, 19 patients agreed to participate (Table 1). The initial idea was to collect data until no new aspects of patients’ experiences were found, but since the experience of the sixth patient differed substantially from the earlier interviewed patients, all 19 patients were interviewed to investigate whether there were any more dissimilar viewpoints.

**Table 1:** Descriptive characteristics of the participants

	Number
Sex	
Men	8
Women	11
Age	
35–44	3
45–54	5
55–64	9
65–70	2
Time in treatment	
10	5
10 + 2	0
10 + 2 + 2	4
10 + 2 + 2 + 2	3
10 + 2 + 2 + 2 + 2 (1 year)	0
2 years of follow-up	0
3 years of follow-up	0
4 years of follow-up	7

*10* means that the patient has completed the 10-day course, *10 + 2* stands for completion of the 10-day course and one 2-day reunion, *10 + 2 + 2* means that the participant has completed an additional 2-day reunion, etc.

### Data Collection

Since the aim was to identify and examine different aspects of patients’ experiences, qualitative interviews were considered to be the most appropriate method for data collection. The reasons for just focusing on one treatment clinic were partly practical, but it was carried out that way to make sure that all informants received the same kind of treatment and to investigate whether there were any variations depending on patient’s treatment time.

Prior the interviews, different aspects, expected to be related to the term “experience of treatment,” were identified and outlined in a mind map based on the first author’s preunderstanding. With the help from the mind map, a semi-structured topic guide was created (Table 2). The informants’ free narrative was the foundation of the interviews, and the interview guide was only used as a support. Time and place for the interviews were chosen by the informants, and each interview that lasted between 20 and 70 min (average 45 min) was recorded on an MP3 player and was immediately transcribed verbatim by the first author to enable an early detection and correction of eventual flaws in the topic guide or interview technique.

**Table 2:** Subject fields in the interview guide

• Life situation
• Experiences of similar treatments
• Experiences of the treatment:
• Being in a group
• Treatment content (dietary advice, exercising, group sessions, lectures, etc.)
• The staff/health professionals
• Telling friends, colleagues, and family about the treatment
• Emotions connected with the treatment and the lifestyle changes
• Thoughts about the future and personal goals

### Analysis

Patient experiences were analyzed from the perspectives of whether the patient expressed themselves as being active contributors in the process of development or as passive recipients and whether their experiences reflected the theory and practice of behavioral medicine or conflicting views. Analysis was conducted according to Malterud’s systematic text condensation [19]. A reflective approach was used throughout the whole analysis process. The analysis consisted of the following steps: (1) repeated readings of transcriptions to obtain a good overview; (2) identifying and coding units of meaning, representing different aspects of patient experience; (3) interpreting similarly coded text units to find patterns; (4) summarizing the content within coded text units to final descriptions, containing the most important aspects of patient experiences; and (5) recontextualizing the final descriptions using the original transcriptions.

### Ethical Aspects

The research design was reviewed and approved by the Research Committee of the Department of Food and Nutrition, Umeå University, and all procedures were conducted in accordance with the ethical guidelines at Umeå University. Information was given that the participation was completely voluntary and that the informants could, at any stage, withdraw their participation. They were also informed that the interviews would be treated confidentially and that they would remain anonymous in all data presentation.

## Results

All informants, regardless of treatment time, had a relatively similar positive experience of the treatment except one whose experience differed radically from the others and therefore is presented separately below.

The sixth informant had an overall negative impression of the treatment and thought it was ineffective and a waste of both time and taxpayers’ money. The informant also thought that obesity had a deeper psychological cause and thereby should be treated with deep, individual therapy or with gastric bypass.

The analysis revealed a process where the informants’ views on lifestyle change were broadened. This broader perspective involved self-development and deepened understanding of own responsibility for health which resulted in new insights about lifestyle change and how to find social support. The process did not seem to be a straight, one-way process but instead a complex interaction affected by the staff, fellow patients, and the individuals’ personal progress. The analysis not only identified many factors in the treatment that could enhance patients’ self-efficacy but it also pointed out things that may hinder it.

### Lifestyle Change—More than Just Improving Your Diet and Exercise

The holistic approach used within the treatment was shown to be of great importance for the informants. Although the majority of the informants initially had focused on their eating and exercise habits, positive changes had appeared in other lifestyle-related areas as well. The opportunity to discuss and work with personal development resulted in new insights for many informants, and several had begun to prioritize their own goals in life. Some had also gained a more balanced view on how to implement and maintain a long-term lifestyle change, e.g., exchanging automatic negative thoughts such as black-and-white thinking for more realistic ones:

“The feeling [the message] - that nothing is wrong, you just do it in a different way … it’s not wrong to fail because you’ve succeeded until the failure… I never thought that way before” IP14, Male

By learning self-reflection and self-monitoring and by gaining knowledge about the process of behavioral change, it was possible for informants to examine their past behavior and plan how to act differently in the future. For example, one woman discovered that she needed to strengthen her self-esteem in order to be able to establish long-lasting lifestyle changes, and another informant had recognized he needed to change his priorities to achieve a balance in life.

Concrete advice about different types of lifestyle choices was experienced as helpful, and informants appreciated that the treatment program included both theory and practice since it made it easier to transform theoretical knowledge into action. Using simple tools, such as pedometers and meal planning forms, was also perceived as an easy way to measure improvement, enhance motivation, and reinforce the feeling of being in control. Informants experienced a treatment free from prohibitions and that there was open attitude towards what kind of lifestyle choices they made:

“I haven’t felt that I had to take it all in [treatment content] or that there was some pressure about it .. It really is like you can pick from a buffet [laugh]” IP1, Female

An essential part of the treatment program was practicing lifestyle-related goal setting, something most informants were unused to and found challenging. One woman had, at first, only vaguely described her goals in writing, but through the goal setting exercises, she learned how to incorporate pictures to create more extensive and emotional descriptions. For her, this creative task was useful to specify and strengthen goals in different areas of life and also leads to insights about her need for personal development.

Several informants expressed a wish for individual treatment in addition to the group treatment. The main reason for this was not only that group treatment sometimes was experienced as being too general but also that informants thought that an overall extended treatment, including both individual and groups sessions, would facilitate lifestyle change.

The staff proved to be a very important part of the treatment, both through their role as professionals and most because of their attitude and personalities. Many informants thought that the staff was genuinely interested in them and in their lifestyle changes, and many experienced the treatment as personal, even though they were in a group:

“Yes, they were five or six who worked there and all [patients] felt that they received a personal treatment from the staff… everyone thought it was something special… and to find a group [the staff] that can radiate that…” IP19, Male

### It Is Up to Me—the Importance of Personal Responsibility

The individual responsibility for good health was something that became clearer during the course of treatment and one woman described it as

“If I ignore it [the need for a lifestyle change], I ignore myself as well” IP8, Female

That the informants’ interest in their own health had grown was also shown by wishes to learn more about the mechanisms behind lifestyle diseases and gain a deeper understanding of the importance of diet and exercise in order to motivate change. Some informants had encountered contradictory advice, particularly regarding diet, and therefore wanted to discuss and know more about the science behind the advice given by the clinic and by media, friends, and relatives.

Increased feelings of confidence and mastery were also expressed, as insights about personal responsibility made informants realize that they, by themselves, could improve their health. However, rapidly improved results due to increased exercise and dietary change could also be experienced as stressful:

“Okay, it’s possible … I’ve seen that I can do it and now I can’t… so that creates a different kind of stress [laughter] … If you don’t know that you can do something about it, it’s easier” IP1, Female

It appeared that it could take a long time to really understand what kind of lifestyle change that was needed and how to do it. A woman, who had been in treatment before, felt it was not until the second treatment she actually realized how to change lifestyle and one informant described lifestyle changes as a lifelong process:

“Since I’m 61, I don’t become slimmer and lighter on my own, and not healthy either… I think these are behavior changes that I have to work with for the rest of my life … Trying to think about what I eat and that I eat at fixed times and … exercise … It’s so darn easy to walk an hour a day… but I don’t do it yet… but I will” IP7, Man

Insights about their own responsibility for health could, however, also entail feelings of shame. This was, for example, revealed when one female informant described how she felt when she had to tell her friends and colleagues about the treatment:

“I thought it was embarrassing, that I needed treatment to lose weight…but I knew, for myself, that it was right… but that others would know about it, that I needed that kind of help, that made me feel a bit sad” IP11, Female

It could be noticed that there were some gender differences since the majority of male informants were completely open about the fact that they were in treatment:

“I’ve told them [co-workers]… If you can restore a car, you should be able to renovate yourself as well” IP9, Man

## I Am Not Alone—Feelings About Participating in Group Treatment

It was obvious that being in a group was perceived as an important aspect of the treatment. Several informants described a special supportive openness within the group which made it easier to share experiences, identify with others, and create distance from one’s own problems. Meeting other patients provided opportunities to talk about things informants did not feel they could talk about with friends or family, and sharing stories could also be perceived as strengthening at a personal level:

“There was a great openness among many… I also tried to be honest… and there were many who tried and it really enriched… Somehow, it built you up, on the inside, to hear it all” IP13, Female

Variation regarding experiences, age, and gender within the treatment group was perceived to have more pros than cons. Some informants felt that the variations were just superficial while others thought that the variation enriched, rather than limited, group sessions. Only one informant experienced the variation as negative and expressed a wish for a more homogenous group. Desires to meet outside of treatment were also expressed. Several informants mentioned that their groups had been talking about seeing each other between meetings but that no one had organized it yet. It was clear that a strong feeling of solidarity had been developed within the initial treatment group, and some informants expressed dissatisfaction about other groups sometimes being invited to the same reunions. The main reason for the dissatisfaction was difficulty in relating to the new group members—which was perceived to limit group discussions. However, and perhaps unfortunately, being in a group could also create group pressure and cause informants to compare themselves with each other:

“I felt that I hadn’t lost anything [body weight]… I thought that I would be, well not laughed at, but that everyone else would have lost a lot of weight and that I would feel really bad among those matchsticks that they had become [laughter] … I had those kinds of thoughts… It wasn’t fun to go back, when I hadn’t performed so to speak… but it was only two who had “performed” [laughter]… so it was not that bad. That made me accept that… well… it is more difficult than I thought” IP1, Female

This fear of failure was also described by another informant. In that case, the informant believed that if she had not lost weight, she would have been too ashamed to go to the reunion. Both informants described thoughts about how others had succeeded but they, themselves, had failed.

## Discussion

This study shows that patients participating in this kind of group-based lifestyle treatment go through a process of self-development which deepens their understanding of own responsibility for health and improves skills on how to find support in others. In all of the three themes presented (the holistic view, personal responsibility, and group treatment), informants expressed themselves in a way that showed them as active contributors to the process of development and not as passive recipients. This is important because the participants must understand that motivation is an everyday ongoing process.

That a holistic treatment approach, which included psychological aspects of lifestyle change, was perceived as important can be seen in relation to the fact that emotions such as stress, shame, and frustration previously have been described as barriers to lifestyle change [16, 17]. An opportunity to learn how to manage the emotions may therefore be crucial to achieve long-term lifestyle change. It also appears to be important to provide useful information and practical tools so that the patients, themselves, can learn how to manage a lifestyle change. Furthermore, the results indicated that the treatment succeeded in its attempt to create favorable conditions for strengthening the informant’s self-efficacy. The components that, according to Bandura, will strengthen self-efficacy were seen in several descriptions of the informants’ experiences [10]. Mastery experience could, for example, be seen in descriptions of how the informants became more confident when they realized they were able to maintain changes and could observe positive results. An unexpected side effect of this was though that if positive results were achieved too rapidly, it could be perceived as stressful since it made informants aware of the possibilities for changing lifestyle. Social persuasion was demonstrated by informants experiencing the staff’s attitude as strengthening and favorable for increasing confidence in their own ability. Stress management was seized upon as helpful as it taught informants how to handle stressful situations as well as reduce internal stress. Seeing other patients succeed functioned as social modeling, but it could also have the opposite effect. If fellow patients did not succeed, it made informants aware of the difficulties in changing lifestyle, which instead could decrease their confidence in succeeding. This highlights the importance of offering a comprehensive treatment program using multiple sources to increase self-efficacy [10].

The staff’s attitude played a crucial role in treatment experience, something that also has been demonstrated earlier [17, 18]. Professionals working with lifestyle modifications are desired by patients to provide both psychological and emotional support [17, 20], and the results confirm that informants experienced such support. The fact that many informants felt they developed a personal relationship with the staff could also be a contributing factor to the positive experience since previously it has been noticed that informal, nonhierarchical relationships between patient and physician have positive effects on both patient satisfaction and treatment outcome [21, 22].

The result shows that patients may experience mixed emotions towards lifestyle treatment. Although the informants felt relieved and satisfied about being in treatment, they could, at the same time, experience feelings of shame. The shame of needing treatment could probably be related to increased insights about the own responsibility for health. In fact, feelings of ambivalence and shame provoked through awareness of personal responsibility have previously been demonstrated in obesity treatment [18]. Shame and embarrassment are strong emotions that have the power to turn off active problem-focused coping and instead involve the patient in passive emotion-focused coping such as defensive avoidance, i.e., denial and rationalization. The reason for this is the high levels of stress and anxiety induced by shame and embarrassment [23]. It may therefore be important to strive for a treatment that includes stress management and is free of preconceptions since further blame probably can reinforce negative feelings and counteract the treatment. Further, there seemed to be some gender differences when it came to feelings of guilt and shame. It was mainly the female informants who felt ashamed of needing treatment while the males had a more open and accepting attitude. This can be seen in relation to previous research which has shown that women generally are more ashamed of their body weight while men, to a greater extent, merely consider it to be a physical state [24]. The fact that the treatment is associated with ambivalence and shame can also be a contributing explanation for the high dropout rate from this kind of treatment [25, 26].

Previous research has shown that group settings are more effective for achieving weight loss than individual treatment [27]. Our study made clear that most informants felt that they could recognize themselves in other patients’ stories and also gain new insights to their own problems by discussing them with others. Furthermore, it should be noticed that strong feelings of safety and solidarity were developed within the initial treatment group which made informants disapprove of having follow-ups with patients from other treatment groups—a fact to keep in mind when planning group-based lifestyle treatments.

Informants’ narratives suggest that they have conformed to the fundamental principles of the theory-based clinical program. Descriptions showed that the participants could analyze the different components of the principles of behavioral change and synthesize them in order to apply them in new settings, which clearly indicates the deeper kind of understanding necessary for long-term behavioral change. However, we must also remember that one informant disputed the whole clinical program and judged it to be a waste of money. All the same, such a statement must be seen as a different but yet well-reasoned opinion on the relationships between behavior, lifestyle, and obesity. Probably, the present clinical program suits most, but not all, people having obesity, and this also highlights the importance of explaining the aims and the content of the program to potential participators before admitting them to the clinic.

Variations in terms of informants’ age, gender, and treatment time may support transferability of the study results. However, exclusion of dropouts from the study and recruitment from only one single clinic need to be considered as limitations. Furthermore, selection was limited by the second recruitment method which only included patients who were present at the treatment clinic during the time period in question. Variation regarding time in treatment was thereby limited to patients who had undergone various parts of the first treatment year or who recently had completed their 4-year follow-up. In light of the generally positive patient experiences revealed in this study on group treatment, it would be of great value to make a comparison with patient experiences of an individual-based treatment of similar kind. It would also be interesting to investigate whether any tangible improvements in self-efficacy actually could be measured among the patients.

## Conclusion

This study shows that lifestyle-focused group treatment in primary and secondary prevention of cardiovascular disease and diabetes serves several important purposes in addition to boosting physiologically measurable parameters. Struggling with lifestyle changes may lead to an increased understanding of own responsibility for health, which can give a sense of relief and lead to an increased ability to take action but may also cause feelings of shame and ambivalence towards treatment. Furthermore, this study shows that it is possible to use a treatment, based on SCT, to stimulate different components that may strengthen self-efficacy, a factor which could be the key for achieving long-term behavioral change.
